# The impact of HLA polymorphism on herpesvirus infection and disease

**DOI:** 10.1007/s00251-022-01288-z

**Published:** 2023-01-03

**Authors:** William H. Palmer, Paul J. Norman

**Affiliations:** 1 Department of Biomedical Informatics, University of Colorado, Aurora, CO USA; 2 Department of Immunology & Microbiology, University of Colorado, Aurora, CO USA

**Keywords:** Human Leukocyte Antigen, Herpesvirus, Immunogenetics, EBV, HCMV, VZV

## Abstract

Human Leukocyte Antigens (HLA) are cell surface molecules, central in coordinating innate and adaptive immune responses, that are targets of strong diversifying natural selection by pathogens. Of these pathogens, human herpesviruses have a uniquely ancient relationship with our species, where coevolution likely has reciprocating impact on HLA and viral genomic diversity. Consistent with this notion, genetic variation at multiple *HLA* loci is strongly associated with modulating immunity to herpesvirus infection. Here, we synthesize published genetic associations of HLA with herpesvirus infection and disease, both from case/control and genome-wide association studies. We analyze genetic associations across the eight human herpesviruses and identify HLA alleles that are associated with diverse herpesvirus-related phenotypes. We find that whereas most HLA genetic associations are virus- or disease-specific, HLA-A*01 and HLA-A*02 allotypes may be more generally associated with immune susceptibility and control, respectively, across multiple herpesviruses. Connecting genetic association data with functional corroboration, we discuss mechanisms by which diverse HLA and cognate receptor allotypes direct variable immune responses during herpesvirus infection and pathogenesis. Together, this review examines the complexity of HLA-herpesvirus interactions driven by differential T cell and Natural Killer cell immune responses.

**Supplementary Information:**

The online version contains supplementary material available at 10.1007/s00251-022-01288-z.

## Human herpesviruses: infection and disease

Herpesviruses are large double-stranded DNA viruses, having a replication cycle characterized by an initial lytic stage, followed by lifelong latent infection that is largely asymptomatic (Adler et al. 2017; White et al. 2012) (Table 1). There are eight human herpesviruses (HHVs), which span three subfamilies: α, β, and γ. These include three α-herpesviruses: varicella-zoster virus (VZV), herpes simplex virus 1 (HSV-1), and herpes simplex virus 2 (HSV-2); three β-herpesviruses: human cytomegalovirus (HCMV), human herpesvirus 6 (HHV-6, often split into HHV-6A and HHV-6B), and HHV-7; and finally two γ-herpesviruses: Epstein-Barr virus (EBV) and Kaposi’s sarcoma-associated herpesvirus (KSHV). HHVs are successful pathogens—with high seroprevalences of most HHVs in most human populations. This success can be partly attributed to intricate mechanisms of immune evasion, host mimicry, and coercion of host signaling pathways that likely underlie their reduced host damage. For example, EBV infects naïve B cells and expresses a B cell receptor homolog that promotes differentiation into the memory B pool, the preferred site of EBV latency (Mancao and Hammerschmidt 2007). HSV-1 induces skin-homing factors in infected T cells, for eventual delivery of virions to peripheral nerves to establish latent infection (Arvin et al. 2010). HCMV encodes multiple Human Leukocyte Antigen (HLA) homologs, including UL18 and UL142, such that downregulation of host HLA cannot be detected by Natural Killer (NK) cells (Beck and Barrell 1988; Wills et al. 2005). While HHVs are considered border-line commensals and can even provide protection during some coinfections (White et al. 2012), they cause or are associated with a multitude of diseases (Table 1) (Goncalves et al. 2017; Griffiths et al. 2015; Mori and Yamanishi 2007; Rabinstein 2017; Taylor et al. 2015; Zerboni et al. 2014) and are likely, on average, detrimental to fitness. Additionally, primate herpesviruses have rarely switched hosts, and the majority of HHVs likely codiverged with humans from great apes (Azab et al. 2018). This antagonistic yet sustained relationship is expected to result in human-HHV coevolution. Indeed, long-term antagonistic coevolution is consistent with the high degree of HHV host specificity, the fine-tuned control of and evasion from the human immune system by HHVs, and the observed genetic variation in susceptibility to HHV infection and disease. Here, we describe the immunogenetic diversity that underlies HLA and HHV coevolution, particularly in relation to the functional consequences of HLA polymorphism in controlling HHV infection.

**Table 1:** Tropism and disease associations of HHV

Virus	Lytic tropism	Latent tropism	Disease associations	Reference
HSV-1 (α)	Oral mucosal epithelium	Sensory neuronal ganglia	Lesions Encephalitis	Rabinstein 2017
HSV-2 (α)	Genital mucosal epithelium	Sensory neuronal ganglia	Lesions Encephalitis	Rabinstein 2017
VZV (α)	Tonsillar epithelium, T cells, skin	Sensory neuronal ganglia	Chickenpox Shingles Postherpetic neuralgia (PHN)	Zerboni et al. 2014
HCMV (β)	Broad tropism, epithelial cells, monocytes	Hematopoietic progenitor cells	Congenital CMV disease Posttransplant CMV disease	Griffiths et al. 2015
HHV-6 (β)	Broad tropism CD4 + T cells, NK cells	Monocytes, macrophages	Exanthema subitum Febrile seizures Encephalitis	Mori and Yamanishi 2007
HHV-7 (β)	Salivary gland epithelium CD4 + T cells	CD4 + T cells, salivary gland epithelium	Exanthema subitum Febrile seizures Encephalitis	Mori and Yamanishi 2007
EBV (γ)	Oral epithelium Tonsillar B cells	Memory B cells	Infectious mononucleosis (IM) Hodgkin’s lymphoma (HL) Nasopharyngeal carcinoma (NPC) Burkitt’s lymphoma (BL) Multiple sclerosis (MS) Hemophagocytic lymphohistiocytosis (HLH)	Taylor et al. 2015
KSHV (γ)	Broad tropism Tonsillar B cells	Memory B cells, monocytes, endothelial cells	Kaposi’s sarcoma (KS) Multicentric Castleman’s disease Peripheral effusion lymphoma KSHV inflammatory cytokine syndrome (KICS)	Goncalves et al. 2017

## The arms of HLA immunity

HLA are cell surface proteins integral in the recognition of pathogens and the subsequent initiation of both cellular innate and adaptive immune responses (Rock et al. 2016). The primary role of HLA molecules is to present small peptide fragments for recognition by the T cell receptor (TCR) on T cells (Boegel et al. 2018; Rock et al. 2016). Polymorphic HLA class I are expressed by most nucleated cells and present intracellular peptides to cytotoxic (CD8+) T cells (Neefjes et al. 2011) and therefore are particularly important in the defense against intracellular pathogens, such as viruses. Additionally, some polymorphic HLA class I serve as ligands for NK cell receptors, most notably the killer cell immunoglobulin-like receptors (KIR) (Pollock et al. 2022) and leukocyte immunoglobulin-like receptors (LILR) (Brown et al. 2004). HLA class I on healthy cells signals “self” through inhibitory NK cell receptors to block NK cell cytotoxicity, but virus-driven HLA downregulation can remove this inhibition, leaving diseased cells vulnerable to NK cell attack (Braud et al. 1998; Hansen and Bouvier 2009; Hilton and Parham, 2017; Pazmany et al. 1996; Shukla et al. 2015). Conversely, activating NK cell receptors complement the function of inhibitory KIR, recognizing HLA class I in a peptide-dependent manner. Thus, activating KIR may promote NK cell cytotoxic responses only during certain infections or alongside loss of other inhibitory signals (Das and Khakoo 2015; Naiyer et al. 2017; Sim et al. 2019). Recent studies have also found that a subset of HLA-DP allotypes (i.e., alleles at the protein level) can interact with the NKp44 NK cell receptor (Niehrs et al. 2019).

HLA class II are canonically expressed by professional antigen-presenting cells, such as dendritic cells, macrophages, and B cells. These molecules can also be upregulated on activated CD4+ and CD8+ T cells. HLA class II proteins bind peptide fragments derived from extracellular pathogens, which have been phagocytosed and subsequently proteolyzed in the endo-lysosomal compartment. Class II presented peptides can be recognized by helper T cells (CD4+), which trigger a wider immune response, most notably including B cell activation (Neefjes et al. 2011).

Through interactions with T cells and NK cells, HLA thus serves as an intersection between innate and adaptive cellular immunity. The molecular pathways governing antigen processing, peptide loading, presentation, and immune responses downstream of HLA have been reviewed in depth elsewhere (Anczurowski and Hirano 2018; Elliott and Williams 2005; Hansen and Bouvier 2009; Kelly and Trowsdale 2019; Neefjes et al. 2011; Rock et al. 2016; Unanue et al. 2016).

## HLA-HHV coevolution

*HLA* loci have long been known to be hyper-polymorphic (Thorsby 2009). For example, the polymorphic *HLA classes I* and *II* have approximately 100 times more alleles than the less-polymorphic *HLA* loci and the presence of *HLA-DRB3*, *HLA-DRB4*, and *HLA-DRB5* varies across individuals (Robinson et al. 2020). These striking patterns have been attributed to host–pathogen coevolution, which can lead to the maintenance of polymorphism in populations (Anderson and May 1982; Lighten et al. 2017; Radwan et al. 2020). Biologically, this argument is supported by observations that distinct HLA allotypes bind an allotype-specific repertoire of pathogen peptides and that peptide-binding sites of HLA harbor the highest levels of polymorphism (Hughes and Nei 1988; Meyer et al. 2018; Parham et al. 1988; Reche and Reinherz 2003). Further, only a subset of HLA allotypes can direct NK cell responses through KIR. Therefore, individuals heterozygous at a given *HLA* locus may be able to defend against a greater pathogen diversity than homozygous individuals, or the benefit of specific alleles may fluctuate alongside pathogen incidence or load (Hughes and Nei 1988; Manczinger et al. 2019; Pierini and Lenz 2018; Takahata and Nei 1990). Throughout human evolution, these natural selective forces have led to the differentiation of *HLA* alleles among populations (Deng et al. 2021), the adaptive introgression of HLA alleles from ancient hominins (Abi-Rached et al. 2011; Greenbaum et al. 2019; Racimo et al. 2015), and ultimately the maintenance of diverse *HLA* alleles.

In the face of a diverse HLA landscape, HHVs have evolved to evade peptide presentation. An early example of this observation was A*11 epitope loss in populations having high A*11 allotype frequencies, consistent with population-specific evolution to evade local T cell responses (de Campos-Lima et al. 1993). More recent population genetic analyses have also found altered patterns of diversity in HLA-binding peptides and T cell epitopes, together suggesting natural selection could favor protein sequences that are less visible to T cells (Palmer et al. 2022; Vider-Shalit et al. 2007, 2009; Wegner et al. 2019). This is particularly evident for proteins involved in the establishment of latency and early lytic infection, which have fewer HLA-binding peptides (Vider-Shalit et al. 2007, 2009), and the remaining HLA-recognized peptides and T cell epitopes have greater protein diversity (Palmer et al. 2022; Santpere et al. 2014) and altered population structure (Palmer et al. 2022; Wegner et al. 2019). Across human populations, EBV latent, but not lytic, epitope frequencies are inversely correlated with frequency of the HLA class I allotype that presents them to CD8+ T cells (Palmer et al. 2022). These patterns highlight the crucial role of T cell responses during the establishment of latency. Evasion of HLA recognition and downstream T cell responses may be required to provide enough time to establish latency, from which a lifetime of reactivation could realize the full transmission potential of infection.

Alongside the many HHV-encoded inhibitors of the peptide presentation pathways (Griffin et al. 2010), these patterns support HHV adaptation to evade HLA peptide presentation. Support for the inverse, whether specific HLA allotypes are specialized in the control of herpesvirus infections, may be gleaned from genetic association studies.

## GWAS highlight a central role for HLA in genetic susceptibility to herpesvirus disease

Genome-wide association studies (GWAS) can provide largely unbiased identification of disease-associated loci. GWAS and case–control studies for herpesviruses have often focused on herpesvirus-associated disease or titers of antibodies targeting herpesvirus genes (Bei et al. 2010; Crosslin et al. 2015; Engdahl et al. 2019; Hammer et al. 2015; Kleinstein et al. 2019; Kuparinen et al. 2012; Rubicz et al. 2013; Sallah et al. 2020; Tang et al. 2012; Tian et al. 2017; Tse et al. 2009). These herpesvirus-focused GWAS reflect three points. First, there are large disparities in research focus across the viruses, with EBV studies representing approximately half of the GWAS and often discriminating among disease phenotypes, while genetic control of beta-herpesviruses and KSHV has only been tested through IgG phenotypes. Second, the genetic variants underlying differential susceptibility to herpesvirus disease are often disease or virus specific. This pattern is formally exemplified in a study that performed 23 GWAS, including those for chickenpox, shingles, HSV-1 cold sore risk, and infectious mononucleosis, where little genetic correlation was observed between herpetic diseases (Tian et al. 2017). Third, and finally, *HLA* is consistently a component of the genetic architecture of herpesvirus complications and immune responses. In all herpesvirus GWAS that have found significant genetic associations (14/17), variants in *HLA* loci have been found significant, and in 12/14 of these, variants at *HLA* were the most significantly associated (Bei et al. 2010; Crosslin et al. 2015; Engdahl et al. 2019; Rubicz et al. 2013; Sallah et al. 2017, 2020; Tang et al. 2012; Tian et al. 2017; Tse et al. 2009). Both HLA class I and class II are represented, with class I associated with viral infection or disease phenotypes and class II associated with circulating IgG levels, consistent with the expected functions of each. For example, EBV IgG levels against EBNA1 are most highly associated with specific variants of *HLA-DRB1* and *HLA-DQA1* (Hammer et al. 2015; Mentzer et al. 2022; Rubicz et al. 2013; Sallah et al. 2017, 2020), while incidence of NPC and IM is associated with certain *HLA-A* and *HLA-B* alleles (Bei et al. 2010; Tang et al. 2012; Tian et al. 2017; Tse et al. 2009). An exception is the antibody response to KSHV, which is associated with a variant located in the genomic interval between *HLA-B* and *HLA-C* (Sallah et al. 2020). Interestingly, the enrichment of *HLA class I* alleles in these associations with disease may reflect, at the population genetic level, the importance of CD8+ T and NK cell responses to herpesvirus infections observed in immunological studies.

Overall, the consistent identification of *HLA* by independent GWAS across herpesviruses is important, as it establishes precedent for HLA in the genetic control of herpesviruses and warrants consideration of the many case–control studies that have been performed with the a priori hypothesis of an *HLA*-herpesvirus association.

## HLA alleles associated with herpesvirus infection and disease

**Fig. 1:**
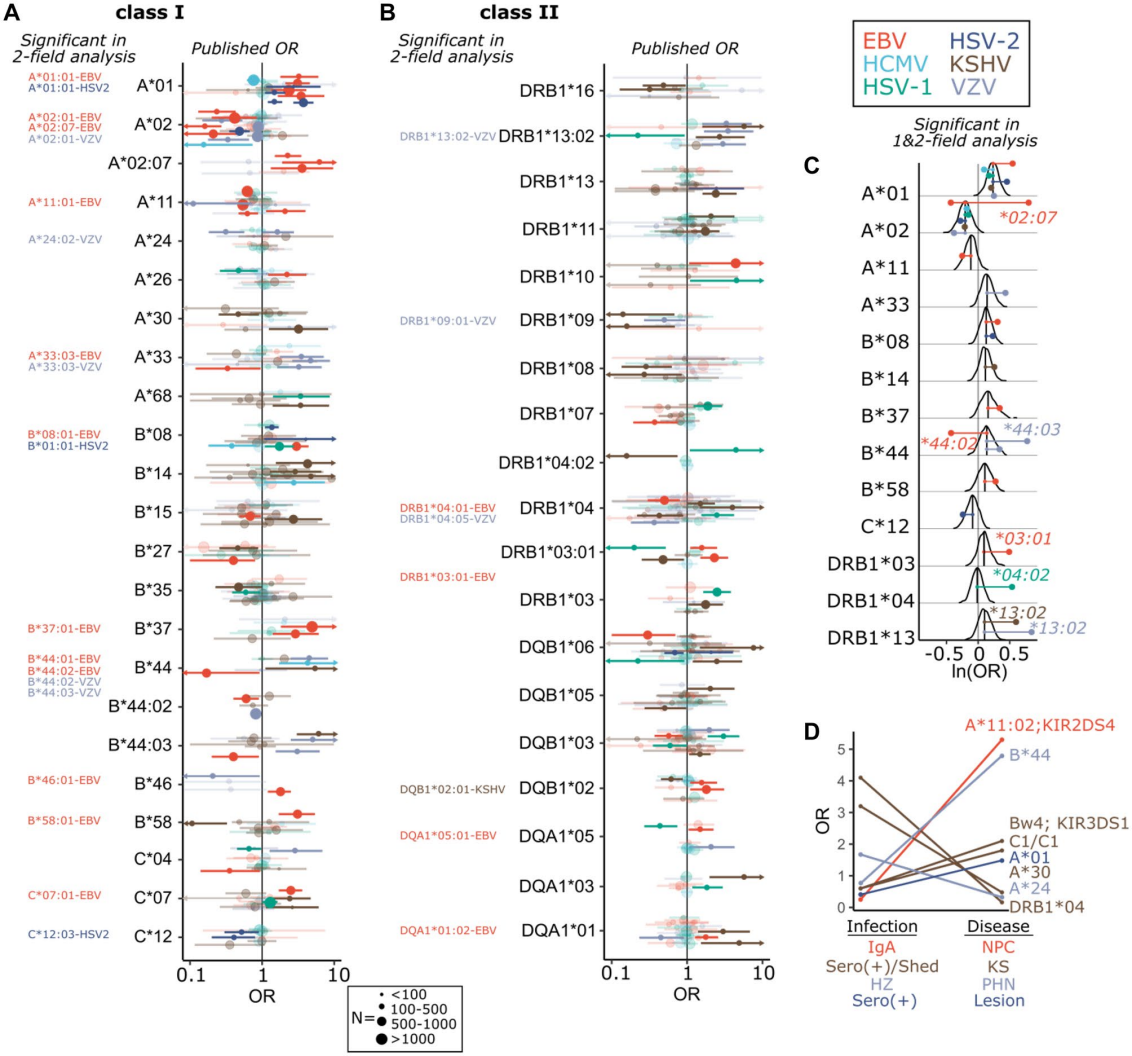
Shared and distinct HLA alleles are associated with herpetic risk. Odds ratios from 38 case/control and GWAS studies were used for a meta-analysis of HLA associations with herpesvirus infection or disease. **A**, **B** Odds ratios (ORs) and 95% confidence intervals are plotted from any *HLA class I* (**A**) or *class II* (**B**) allele with at least two significant associations. Non-significant associations are faded, while significant associations are bold (as determined from *p* values reported in source publication). Arrows indicate the confidence intervals extend through the bounds of the plot. Points are sized according to the number of participants in each study. Two analyses were performed on published ORs: using only two-field allele data or using both one and two-field data. Significant HLA:virus phenotype associations from two-field only data are provided in the margin in **A**, **B**. **C** Model output from the meta-analysis of herpesvirus-HLA associations using one and two-field data. For each allele having a significant association with at least one virus, the posterior probability distribution of the log odds ratio is plotted, estimated from association with all measured herpesvirus phenotypes. Colored lines and points show the estimated effect size of the individually significant virus-specific genetic associations. Associations specific to an allele at two-field resolution are indicated. All virus-specific effects are shown for A*01 and A*02, each of which had significant general associations across herpesviruses. **D** A subset of alleles differentially impacts herpesvirus infection or disease. Shown are specific genotypes (right) that have significant, but opposite, impacts on herpesvirus infection and subsequent disease. Measured infection (i.e., IgA seropositivity, shedding, and herpes zoster (HZ)) and disease (NPC, KS, and PHN) phenotypes are shown below the plot and colored by virus. Genotypes with a semicolon are joint KIR/HLA genotypes. C1 and Bw4 are HLA ligands that interact with KIR

Associations between specific *HLA* alleles and herpesvirus diseases were described both before and alongside the discovery of HLA molecular function (Lipinski et al. 1979; Lu et al. 1990; Russell and Schlaut 1975; Simons et al. 1975; Stern 1978). Since then, a multitude of case–control studies have been performed, often focusing on European populations or populations with an unusually high rate of disease. For examples of the latter, EBV-associated nasopharyngeal carcinoma (NPC) is prevalent in Southern Chinese, Taiwanese, and North Africans, Kaposi’s Sarcoma (KS) has a high incidence in Sardinia, and HSV shows high prevalence in sub-Saharan African and South/Central America (Bei et al. 2012; Cottoni et al. 2004; Samandary et al. 2014). These studies have identified specific *HLA class I* and *class II* alleles associated with herpesvirus infection and disease severity and generally mirror the patterns identified in GWAS. Of particular interest are those alleles that have been found associated with herpesvirus infection or disease by multiple independent studies, across multiple populations, or across multiple herpesviruses (Fig. 1A, B).

To focus our review on the most reproducible evidence, we compiled 38 datasets that tested for associations between *HLA* alleles and herpesvirus-related phenotypes and performed a pan-herpesvirus meta-analysis of *HLA* associations. GWAS or HLA-genotyped case–control studies were identified through a literature search for the name of each virus species and either “HLA” or “Human Leukocyte Antigen.” Studies were included in the analysis if they provided either odds ratios (OR) with confidence intervals (CIs) or the appropriate raw frequency data that we could use to calculate OR and CIs. The final dataset included four genome-wide association studies (GWAS) that genotyped HLA to at least first-field resolution (three by imputation and one by PCR-SSOP) (Bei et al. 2010; Tang et al. 2012; Tian et al. 2017; Tse et al. 2009) and 34 candidate gene case/control studies. We recorded ORs and their 95% CIs from all alleles genotyped in each study, totaling 1358 published measurements. A small subset of studies measured two phenotypes (Gayà et al. 2004; Goedert et al. 2016; Lekstrom-Himes et al. 1999; Sato-Takeda et al. 2004), for example, Sato-Takeda et al. (2004) measured both herpes zoster (HZ) and postherpetic neuralgia (PHN) for the same individuals (Sato-Takeda et al. 2004). To avoid pseudoreplication, we used only one phenotype and their respective associations, chosen based on the strongest HLA association.

Of the 38 studies, 26 genotyped HLA at two-field resolution. We analyzed these two-field data in two ways. First, we analyzed only the studies that genotyped HLA at two-field resolution, removing lower resolution associations (Fig. 1A, B “2-field analysis”). Second, we analyzed all one and two-field associations together (Fig. 1C “1 and 2-field analysis”). To reduce the final model complexity, we performed a preliminary analysis to identify the two-field resolution alleles that have significantly different odds ratios than expected from first-field resolution typing. These analyses identified HLA-A*02, B*44, DPB1*04, DQA1*03, DRB1*03, DQB1*03, DRB1*13, DRB1*04, and DRB1*11 as having significant differences in associations at the two-field resolution, and their respective two-field alleles were included in the final analysis. We used MCMCglmm (Hadfield 2010) to model the log odds ratio of HLA-HHV associations using a random effects model with predictors associated with one-field genotypes, two-field genotypes, and the interactions of each with different HHVs. The standard error for each odds ratio was also included as a random effect to model the sampling variance. We assess significance of each allele, for each virus and across viruses, by assessment of the proportion of iterations the Markov chain is positive or negative (Palmer et al. 2018). We include all alleles identified as significant by two or more studies, independent of our model, in Fig. 1A, B, and discuss those results significant from the 1 and 2-field analysis in the following sections. All published associations can be found in Table S1.

First, we tested whether any *HLA* allele defined at one-field is broadly associated with herpetic infection and disease across all HHVs. We found evidence that A*01 and A*02 allotypes are generally associated, respectively, with susceptibility (random effects model; MCMCp = 0.016) and resistance (random effects model; MCMCp = 0.03) to HHV infection and disease (Fig. 1C). In European populations, A*01 is consistently the most significant HLA susceptibility factor for EBV + HL and IM (Hjalgrim et al. 2010; Huang et al. 2012b; Johnson et al. 2015; Niens et al. 2007) is significantly associated with HSV-1 shedding and lesion rate (Lekstrom-Himes et al. 1999) and with HSV-2 lesion rate (Magaret et al. 2016). Conversely, A*01 is found to be associated with lower risk of HCMV reactivation in solid organ transplant donors in an Irish cohort (Hassan et al. 2016), although this is not the case in an Ashkenazi Jewish cohort (Parks et al. 2018). A*02 is well replicated to be protective against infectious mononucleosis (IM) and Hodgkin’s lymphoma (HL) (Hjalgrim et al. 2010; Huang, Kushekhar et al. 2012; Niens et al. 2007) and also significantly associated with a lower risk of chickenpox (Tian et al. 2017), shingles (Tian et al. 2017), PHN (Meysman et al. 2015), and HCMV reactivation in glioma patients (Han et al. 2017). A*02 carriers also have lower titers of antibodies that recognize HHV6A, which could be caused by more efficient clearance of infected cells by CD8 + T cells (Engdahl et al. 2019; Hammer et al. 2015). Notably, A*02 is present at moderate frequency in most populations (Gonzalez-Galarza et al. 2020). Specifically, protection is more often associated with the A*02:01 allele (Jones et al. 2016; Tian et al. 2017), whereas the A*02:07 allele is a risk factor for EBV-mediated NPC and HL in Chinese and Taiwanese populations (Hildesheim et al. 2002; Huang et al. 2012a; Su et al. 2013; Tse et al. 2009). The single amino acid difference (Y99C) between A*02:01 and A*02:07 occurs in the peptide binding pocket and has been shown to impact the efficiency of peptide presentation from the EBV latent protein, LMP2, suggesting differential coordination of the CD8 T cell response could underlie NPC protection (Lee et al. 1997). Other explanations include strong interactions between A*02 and an unknown environmental variable or functional epistasis with other population-specific variants.

Next, we highlight HLA alleles with supported associations that are individual to or variable across HHVs (Fig. 1C). B*08 mirrors A*01 associations. Although both allotypes are part of the long-range A1-B8-DR3-DQ2 haplotype found at high frequency in Europeans, the B*08 association with HL risk remains after accounting for A*01 (Johnson et al. 2015). The presence of B*44 is associated with either greater or lesser disease risk, depending on the HHV. For example, it is a risk factor for PHN (VZV) in Japanese populations (Meysman et al. 2015), protective against NPC (EBV) in Taiwanese (Hildesheim et al. 2002), protective against shingles (VZV) and HL (EBV) in Europeans (Johnson et al. 2015; Tian et al. 2017), associated with increased risk of KS (KSHV) in Cameroon (Cornejo Castro et al. 2019; Goedert et al. 2016; Guech-Ongey et al. 2010) and with HCMV reactivation in Iran (Futohi et al. 2015). The two most frequent B*44 alleles are B*44:02 and B*44:03, which differ by a single amino acid and can direct differential CD8 T cell responses (Herman et al. 1999). Notably, B*44:03 is the dominant allele in Japanese and Cameroonian populations where B*44 is associated with HHV risk, with B*44:02 close to absent in these populations. Analyses with two-field genotyping have identified B*44:02 as significantly associated with a decreased risk in EBV infection and B*44:03 with an increased risk during VZV infection (Fig. 1C). For *class II* genes, a haplotype with DRB1*13:02 and DQB1*06:04 is associated with susceptibility to KS in AIDS patients in the US and Italy (Dorak et al. 2005; Guerini et al. 2006; Masala et al. 2005), with PHN in Japan (Meysman et al. 2015), and a similar haplotype (DRB1*13:01 and DQB1*06:03) associated with higher IgG levels to HHV-6A (Engdahl et al. 2019). DRB1*13 alleles are also associated with higher risk of HSV-2 lesions (Lekstrom-Himes et al. 1999), while DRB1*13:02 is associated with lower risk of HSV-1 lesions (Malo and Wank 1998), highlighting the impact of a few residue differences on disease risk. Finally of note, while A*11 is only identified as significant in the meta-analysis in its well-defined association with decreased NPC (EBV) risk in South Asian populations (Bei et al. 2010; Hildesheim et al. 2002; Tang et al. 2012; Tse et al. 2009), it has also been found associated with KS in Italians (Goedert et al. 2016) and HCMV reactivation in transplant patients (Chen et al. 2001).

Intriguingly, a subset of genotypes was observed to have significant but opposite impact on HHV infection versus disease phenotypes (Fig. 1D). A compound genotype encoding KIR2DS4 and its ligand A*11:02 is observed to be associated with reduced EBV/IgA/VCA seroconversion, a risk factor for NPC, but with increased risk of developing NPC (Gao et al. 2017, 2015). A*24 and B*44 are associated with increased (Sato-Takeda et al. 2004) and decreased (Tian et al. 2017) risk of VZV reactivation, respectively, but when the VZV complication, PHN, is considered, the inverse is found (Ozawa et al. 1999; Sato et al. 2002; Sato-Takeda et al. 2004; Sumiyama et al. 2008). A joint genotype encoding KIR3DS1 and its putative Bw4 ligand are associated with reduced KSHV seropositivity, but increased risk of KS (Goedert et al. 2016). Inverse genetic associations with KSHV infection and development of KS were also observed in the context of A*30 (Goedert et al. 2016; Masala et al. 2005), DRB1*04 (Alkharsah et al. 2007; Dorak et al. 2005), and homozygosity of HLA-C1 allotypes (Caselli et al. 2014; Goedert et al. 2016; Guerini et al. 2012). Finally, A*01 is associated with a reduced HSV-2 (Lekstrom-Himes et al. 1999) seropositivity rate but increased occurrence of lesions (Magaret et al. 2016). An alluring hypothesis is that these observations could represent immunological trade-offs, whereby protection against infection has pathological consequences.

## HLA alleles and their receptors coordinate variable immune responses

Variation in HLA-mediated immunity is largely described as differential CD8+ T and NK cell responses to infection, broadly aligning with the known importance of these cells in immune responses to herpesviruses (Dittmer and Damania 2016; Egan et al. 2013; Laing et al. 2018; Paludan et al. 2011; Picarda and Benedict 2018; Smith and Khanna 2013; Taylor et al. 2015). CD8+ T cells recognize peptides presented by HLA, and therefore, the distinct viral peptides bound by specific HLA allotypes directly alter T cell recognition (Jing et al. 2016; Sylwester et al. 2005; Taylor et al. 2015). NK cell receptors, some highly polymorphic, recognize infected cells through the combinatorial engagement of ligands, including HLA, on a target cell (Guethlein et al. 2015; Hilton and Parham 2017; Vivier et al. 2011). Allotype-specific peptide presentation by HLA can coordinate variable T cell responses, leading to variation in disease outcome following herpesvirus reactivation. Thus, variable NK cell responses can be directed by allotype-specific engagement of HLA by NK cell receptors. In some cases, there are described immunological mechanisms consistent with and possibly driving the observed HLA genetic associations with HHV phenotypes. Thus, for each HHV subfamily (*α*, *β*, and *γ*), we briefly describe the role of HLA in the immune response to HHVs and then focus on experimental data that lend support to genetic associations described in population studies (i.e., case/control and GWAS).

*α-Herpesviruses*. The genomes of HSV-1, HSV-2, and VZV share a high proportion of orthologues, likely explaining why the viruses induce cross-reactive antibody (Schmidt et al. 1969) and T cell responses (Jing et al. 2016; Laing et al. 2018; Ouwendijk et al. 2013). CD8 and CD4 T cells control HSV-1 and HSV-2 infection and can be observed surrounding latently infected ganglia (Ouwendijk et al. 2013; van Velzen et al. 2013; Zhu et al. 2007). However, the CD4 T cell response is considered most important during VZV infection (Haberthur et al. 2011; Laing et al. 2018) and its magnitude is associated with less severe HZ (Weinberg et al. 2009).

Proteome-wide CD4 and CD8 T cell screening for HSV-1, HSV-2, and VZV antigens has identified individual and sometimes HLA-restricted responses. While these studies have confirmed the relevance, breadth, and interindividual variation of CD4+ T cell responses to HSV-1 and VZV (Jing et al. 2012; van Velzen et al. 2013), we did not observe overlap between any *HLA class II* associations with α-herpesvirus disease (Fig. 1) with any obvious trends in class II-restricted T cell responses. However, CD8+ T cell screens have identified variation in HLA class I-restricted T cell responses that overlap with the class I associations found in case–control studies. In two genome-wide screens for HSV-1 and HSV-2 T cell antigens, B*44 allotypes tended towards weak CD8 T cell responses (Jing et al. 2012; Koelle et al. 2003; van Velzen et al. 2013). While no direct associations with HSV-1 and these alleles have been found, the A*33:03-B*44:03-DRB1*13:02 haplotype is associated with PHN (Sato et al. 2002; Sato-Takeda et al. 2004). Also, A*02, associated with lower risk of PHN, is predicted to bind approximately seven times more high-affinity peptides from VZV than B*44 (Meysman et al. 2015) and mediates protective CD8 T cell responses against ocular herpes (Dervillez et al. 2013; Srivastava et al. 2015). Similar to B*44, three HLA-C allotypes including C*04:02 were found to generate weaker CD8 T cell responses (Jing et al. 2012), and C*04 allotypes are associated with increased risk for symptomatic HSV-2 infection (Lekstrom-Himes et al. 1999). Taken together, subpar CD8 T cell responses generated by B*44 and C*04 allotypes could underlie genetic associations between these alleles and α-herpesvirus pathogenesis.

*β-Herpesviruses.* Interestingly, GWAS for HCMV IgG levels did not find significant genetic associations (Hammer et al. 2015; Kuparinen et al. 2012) and ex vivo proteome-wide epitope mapping did not observe differences in the CD4 or CD8 T cell response across diverse HLA haplotypes (Sylwester et al. 2005). However, solid organ transplant patients with A*01, A*02, or B*44 had higher numbers of HCMV-specific activated T cells (Fernández-Ruiz et al. 2015), and A*01 and A*02 are associated with reduced HCMV seropositivity and reactivation, respectively (Han et al. 2017; Hassan et al. 2016), suggesting differential CD8 T cell responses could drive transplant outcomes in an HLA-dependent manner.

HCMV case–control studies have primarily focused on epistatic genetic variation between NK cell receptors and their HLA ligands as underlying HCMV disease susceptibility, most often in the context of transplantation. This focus is supported by HCMV biology—HCMV infection induces a striking reorganization of the NK cell compartment, with NKG2C^+^, KIR^+^ NK cells proliferating following initial infection or HCMV reactivation, leaving stable imprints on the NK cell repertoire that can last over two years (Béziat et al. 2013; Gumá et al. 2004, 2006; Rölle et al. 2014). These NK cells are likely effective in recognizing HCMV-infected cells, as they readily produce IFNγ (Foley et al. 2012) and exhibit antibody-dependent cellular cytotoxic lysis of infected fibroblasts (Costa-Garcia et al. 2015).

NKG2C is an NK cell-activating receptor that recognizes HLA-E, which is a moderately polymorphic HLA class Ib. HLA-E presents peptides derived from HLA class I leader sequences but can also present peptides derived from the UL40 protein of HCMV (Jouand et al. 2018; Pietra et al. 2003; Tomasec et al. 2000). The UL40 peptide is hyper-variable as compared to the rest of the protein, with diversity peaking at a residue that binds to NKG2C, and some UL40 variants fail to provoke a strong adaptive NK cell response (Hammer et al. 2018; Heatley et al. 2013; Vietzen et al. 2021). HLA-E also interacts with the inhibitory NK cell receptor, NKG2A, suggesting HCMV-mediated stabilization of HLA-E and UL40 diversification may reflect a trade-off between evasion of and susceptibility to NK cell damage mediated by NKG2A and NKG2C, respectively (Vietzen et al. 2021). The importance of these differential NK cell responses is demonstrated by genetic associations between UL40 and HLA-E variants with transplant outcomes (Guberina et al. 2018; Vietzen et al. 2021).

Whereas interference with NKG2C or HLA-E on NK cells or infected fibroblasts, respectively, can reduce NK cell proliferation (Rölle et al. 2014), KIR+ NK cells from NKG2C-null individuals still proliferate in response to HCMV infection. This observation suggests additional involvement from KIR and their HLA ligands (Della Chiesa et al. 2014). Indeed, numerous case–control studies have examined the impact of KIR repertoire on HCMV infection following solid organ or hematopoietic cell transplants. Most of these studies find a protective effect of activating KIR on HCMV reactivation, from the donor in the case of hematopoietic transplant or in the recipient during solid organ transplant (Cook et al. 2006; Gonzalez et al. 2014; Hadaya et al. 2008; Stern et al. 2011; Zaia et al. 2009). This protection by activating KIR has been confirmed during primary HCMV infection in immunocompetent hosts, outside the context of immunosuppressed transplant patients (Di Bona et al. 2014). However, other case–control studies find specific combinations of KIR alongside the HLA-C1 ligand to be protective against primary HCMV infection following transplant from a seropositive donor (Jones et al. 2014; van Duin et al. 2014). These findings are broadly reflected in the immunological data: NK cells expressing both activating and inhibitory KIR proliferate in response to HCMV infection (Béziat et al. 2013; Della Chiesa et al. 2014). The activating KIR, KIR2DS1, can directly recognize HCMV infection and aids decidual NK cells in the clearance of HCMV-infected placental cells (Crespo et al. 2016; van der Ploeg et al. 2017). Proliferation of NK cells expressing inhibitory KIR is dependent on NK cell education and is observed only in individuals with allotypes of inhibitory KIR and HLA that can interact (Charoudeh et al. 2013; Djaoud et al. 2013; Manser et al. 2019). Thus, the variable associations in case–control studies may reflect specific gene content or gene-by-environment interactions, as individual KIR haplotypes can contain 0 to 5 activating KIR with variable presence of inhibitory KIR and individual HLA haplotypes can encode a variable number of KIR ligands.

LILRB1 is an inhibitory immune cell receptor that binds broadly to HLA class I ligands (Cosman et al. 1997), and specific variants have also been associated with HCMV infection following transplantation (Yu et al. 2018). To exploit LILRB1 and other inhibitory receptors that recognize class I ligands, HCMV encodes an MHC class I homolog, UL18 (Beck and Barrell 1988), which has been shown to inhibit LILRB1-expressing NK cells (Cosman et al. 1997; Prod’homme et al. 2007). LILRB1-binding domain polymorphism underlies functional variation in the ability to interact with classical HLA class I and UL18 (Yu et al. 2018), but not with HLA-G, where this interaction has a role in placentation (Apps et al. 2007). Murine cytomegalovirus also encodes an MHC-I mimic (Farrell et al. 1997), suggesting that the altering of NK cell responses is critical for infection by cytomegaloviruses.

Although other β-herpesviruses, HHV-6A, HHV-6B, and HHV-7, have not been studied as extensively as HCMV, NK cells seem similarly important in the immune response mounted during their infection (Atedzoe et al. 1997; Flamand et al. 1996), and HHV-6 is both T- and NK-tropic (Eliassen et al. 2017; Lusso et al. 1993). Possibly reflecting functional variation of HLA and KIR, NK cell clones within and across individuals have vastly different cytolytic ability against HHV-6-infected targets (Malnati et al. 1993). Supporting an important function for HLA/KIR interactions during HHV-6A infection, a genotype containing HLA-C1 and its cognate receptors KIR2DS2 and KIR2DL2 is associated with susceptibility to HHV-6A infection (Rizzo et al. 2019). In total, more case–control, genome-wide association, and functional immunity studies will be required to understand the role of HLA variants in infection by these *β*-herpesviruses.

*γ-Herpesviruses.* An important role for the cytotoxic T cell response during EBV infection is supported by the large expansion of EBV-reactive CD8 + T cells that defines IM, their sometimes diminished response in HL and NPC patients (Fogg et al. 2009; Gandhi et al. 2006; Li et al. 2007), and strong HLA class I associations localizing to the residues of the peptide-binding groove. During both primary infection and IM, CD8 + T cells against highly immunogenic lytic protein epitopes abound, followed by an immunodominant response towards EBNA3 proteins during the switch to latency. However, while EBNA3 expression is observed in EBV complications of the immunosuppressed (e.g., HIV-associated or posttransplant lymphoproliferative disorders), they are not often expressed in latently infected B cells, nor in HL or NPC malignancies (Price and Luftig 2015). Subdominant T cell responses towards latent proteins EBNA1, EBNA2, and LMP2 also occur, can be HLA-restricted, and may reflect HLA genetic associations with disease (Blake et al. 1997; Brooks et al. 2016; Khanna et al. 1998; Lee et al. 1993). For example, a GWAS identified the peptide-binding grooves of B*38:02 and B*55:02 associated with susceptibility and resistance to NPC, respectively (Tang et al. 2012), and B*38:01 and B*55:01 have been found unique in orchestrating immunodominant responses to EBNA2 (Brooks et al. 2016). Additionally, A*01, A*02, and A*11 have some of the most consistent associations with EBV-associated HL and NPC. Numerous A*02 and A*11 presented peptides from EBV latent proteins stimulate CD8+ T cells, whereas none have been described for the susceptibility allele, A*01 (Taylor et al. 2015). For example, cytotoxic T cell expansions directed against LMP2A are greater from A*02+ donors than A*02− donors in EBV+ HL, but not EBV − HL (Jones et al. 2016). Consistent with these findings, peptide prediction software finds approximately tenfold more high affinity EBNA1, LMP1, and LMP2 peptides presented by A*02 than A*01, whereas this was not observed for other sets of length-matched EBV lytic proteins. While there has been considerably less effort in discovering KSHV epitopes presented by different HLA allotypes (Fang et al. 2019), the current evidence suggests A*02 could be similarly protective (Ribechini et al. 2006; Robey et al. 2010).

NK cells also mediate protection against EBV disease (Chijioke et al. 2016). In response to lytic EBV infection, an early differentiated NK cell subset (CD56^dim^, NKG2A^+^, KIR^−^) proliferates in peripheral blood, protects against both infection and IM, and persists (Azzi et al. 2014; Chijioke et al. 2013; Hendricks et al. 2014; Pappworth et al. 2007; Williams et al. 2005). A similar subset of dendritic cell-activated IFNγ^high^ NK cells (CD56^bright^, NKG2A^+^) is found in tonsils and more potently restricts EBV transformation of B cells (Jud et al. 2017; Lünemann et al. 2013; Strowig et al. 2008). While the exact role for KIR during EBV infection and disease remains unclear, disconnected observations support the hypothesis of KIR involvement. A peptide presented by HLA-C2 allotypes during EBV infection is recognized by the activating KIR2DS1, tonsillar EBV-responsive NK cells express KIR, and acute EBV infection is associated with variable shifts in the percentage of mature NK cells that express KIR (Hendricks et al. 2014; Lünemann et al. 2013; Stewart et al. 2005). Further, multiple candidate gene case/control studies have found associations between KIR polymorphism and EBV infection and complications (Besson et al. 2007; Durovic et al. 2013; Gao et al. 2015; Huo et al. 2015; Kovacic et al. 2005; Qiang et al. 2012). Although not altogether consistent in associations, these studies tend towards the finding that activating KIR underlie susceptibility to NPC, BL, and EBV-HLH (Huo et al. 2015; Kovacic et al. 2005; Muriuki et al. 2021; Nowak et al. 2019; Qiang et al. 2012), although protective against HL (Besson et al. 2007; Jiang et al. 2022). Interestingly, while the A*11:01 allotype is protective from NPC, A*11:02, which differs by one amino acid that results in productive KIR binding, is not and is associated with NPC in a KIR-dependent manner (Gao et al. 2017, 2015; Graef et al. 2009). Similar to EBV, NK cells respond to KSHV and KS (Caselli et al. 2014; Goedert et al. 2016; Guerini et al. 2012), but the only suggestion of a role for KIR are genetic association studies (Beldi-Ferchiou et al. 2016; Dupuy et al. 2012; Sirianni et al. 2002). Together, these genetic associations indicate a function for KIR + NK cells during γ-herpesvirus infection and pathogenesis of malignant disease.

Finally, and unique to EBV, HLA class II serves as an EBV entry receptor into B cells (Li et al. 1997). HLA-DQ allotypes vary in their binding affinity for the EBV envelope glycoprotein, gp42, where glutamic acid at residue 46 of DQB1 is required for gp42 interaction and EBV entry (Haan and Longnecker 2000; McShane et al. 2003). As such, DQB1*03 homozygotes are resistant to EBV infection through the DQ heterodimer (Haan and Longnecker 2000; McShane et al. 2003). However, EBV infection of individuals with non-permissive HLA-DQ allotypes could conceivably proceed through other class II HLA molecules. Consistent with these molecular differences, HLA-DQB1*02 allotypes are associated with gp42 binding and increased EBV seropositivity whereas HLA-DQB1*03, *04, *05, and *06 alleles associate with lack of seroconversion and less strong binding affinity (Li et al. 2017). Following infection, gp42 is repurposed as an HLA class II evasion protein to dampen the CD4 + T cell response (Ressing et al. 2003). While the role of HLA-DQB1 allotypic diversity in immune evasion has not been determined, a failure of gp42 to bind certain HLA-DQ allotypes could promote CD4+ T cell activation, leading to the observed association of specific HLA-DQB1 variants with seropositivity.

## Concluding remarks

HLA undoubtedly is under unique natural selective pressures, driven in part by the role in protection from diverse pathogens, resulting in the maintenance of genetic variation that impacts immune responses. The association of HLA variation with HHV pathology is an imperfect window into their sustained coevolutionary relationship. In this complex scenario, diverse HLA alleles are associated with HHV infection and disease differentially across populations. A*01 and A*02 may underlie more general genetic differences in HHV control, but these allotypes also among the most well studied, which could suggest hidden ascertainment biases. A better understanding of HLA-HHV coevolution and the impact of HLA on HHV disease will be gained from association studies from under-represented populations and the continued functional dissection of observed disease associations.

## Supplementary Information

Below is the link to the electronic supplementary material.

Supplementary file1 (XLSX 96 KB)
